# Establishment of reference ranges and stability assessment for target height completion rate based on the China Health and Nutrition Survey (CHNS)

**DOI:** 10.3389/fpubh.2026.1931404

**Published:** 2026-09-14

**Authors:** Ying Zhao, Jialin Mu, Ketong Guo, Jinhan Huo, Hui Zou

**Affiliations:** 1 Jinan Maternity and Child Care Hospital Affiliated to Shandong First Medical University, Jinan, China; 2 Shandong First Medical University, Jinan, China; 3 Chengde Medical University, Chengde, China

**Keywords:** CHNS, genetic target height, height SDS, tanner method, target height completion rate

## Abstract

**Background:**

Height SDS is a well-established and effective screening indicator for clinical growth assessment. Nevertheless, as a purely population-relative metric, it does not in isolation incorporate parental height information. To complement the conventional SDS-based evaluation, we propose the Target Height Completion Rate (THCR = adult height/genetic target height × 100%), an index additionally adjusted for parental genetic height. Based on a large population-based cohort from the China Health and Nutrition Survey (CHNS), this study aims to establish population-level reference ranges of THCR in general Chinese adults and systematically evaluate its cross-subgroup stability, to provide a baseline benchmark for clinical THCR evaluation and future disease cohort research.

**Methods:**

Data were drawn from the China Health and Nutrition Survey (CHNS, 1989–2015), yielding 5,098 adult individuals (≥18 years; 3,122 males, 1,976 females) with matched parental height records. Genetic target height was calculated using the Tanner method [males: (father's height + mother's height + 13)/2; females: [father's height + mother's height – 13]/2]. After calculating THCR, its associations with sex, birth cohort, urban-rural residence, educational attainment, and other factors were evaluated, and percentile reference ranges were established.

**Results:**

THCR exhibited an approximately normal distribution, with a mean of 102.19% (SD 3.25%) and a median of 102.18%. Female THCR (102.99% ± 3.28%) was higher than male THCR (101.69% ± 3.13%) by 1.30 percentage points. Showed a weak positive correlation with THCR (low education group 101.87% → high education group 102.67%), and the univariable urban-rural difference (0.30 pp; urban 102.38% vs. rural 102.08%, *P* = 0.002) was attenuated and no longer significant after adjustment for educational attainment (+0.14 pp, *P* = 0.156), indicating that it largely reflected the higher educational attainment of urban residents.

**Conclusions:**

THCR in the general Chinese population remained stably centered around 102%, with minimal between-subgroup differences (all < 1.5 percentage points across cohorts from the 1950s onward). The percentile distribution established in this study provides a baseline reference for future disease cohort studies.

## 1 Introduction

### 1.1 Limitations of existing height evaluation indices

Height is one of the core indicators reflecting growth, development, and nutritional status. In clinical practice, the height standard deviation score (height SDS) is the measure most commonly used to evaluate individual height. It is calculated as SDS = (measured height – mean height of the same-age and same-sex reference population) / population standard deviation, representing the individual's relative position within the general population. The World Health Organization (WHO) laid the foundation for SDS application through its global child growth standards based on healthy breastfed children from multiple countries (1). Contemporary international research on clinical short stature likewise employs SDS as the evaluation criterion (2); SDS thus occupies a central position in both clinical practice and disease research.

A fundamental limitation of SDS, however, is that it merely compares an individual against a population average without accounting for genetic background. Two children with identical measured height—one from a short-statured family (father 155 cm, mother 150 cm) and the other from a tall-statured family (father 180 cm, mother 170 cm)—would receive the same SDS, yet the clinical implications of their growth potential would be markedly different. Under the SDS framework, the former might be classified as “familial short stature with normal development,” whereas the latter might represent “a clear failure to reach genetic growth expectation.” SDS cannot distinguish between these two scenarios.

This deficiency is especially consequential in research on the impact of disease on height. Studies of the effects of conditions such as chronic kidney disease (CKD) (3), congenital heart disease (CHD) (4), and inflammatory bowel disease (5) on height predominantly employ SDS as the outcome measure. In the Chinese context, the evidence-based guideline for the diagnosis and treatment of growth impairment in pediatric CKD, issued by the Nephrology Group of the Pediatric Society of the Chinese Medical Association (6), uses height SDS < −1.88 as the diagnostic threshold for growth impairment, and treatment evaluation within this guideline likewise centers on changes in height SDS as the core indicator. However, because SDS is calculated solely against a population mean and does not in isolation incorporate parental height information, its use alone does not directly separate height deficits caused by disease pathology from those resulting from familial short stature; this distinction requires reference to the individual's genetic target height derived from parental heights. For the general population, the magnitude to which individual height realizes parental genetic potential also lacks standardized population-level reference data.

This methodological gap directly hinders the precise evaluation of growth therapeutic efficacy and the implementation of personalized growth management. Therefore, developing a genetic background-adjusted growth indicator and establishing its normative reference range is a critical prerequisite for quantifying disease-related growth impairment and advancing individualized precision medicine in pediatric endocrinology and developmental health.

### 1.2 Clinical application of the tanner method and the target height completion rate

Genetic target height is the expected adult height of an individual estimated from the heights of their biological parents—first proposed by Tanner et al. in 1970 (7). The Tanner formula is:

Male target height (cm) = (father's height + mother's height + 13) / 2.

Female target height (cm) = (father's height + mother's height – 13) / 2.

Previous research has confirmed the validity of the Tanner method at the population level. Luo et al. validated the applicability of this formula in a large-scale study involving 2,402 children and noted that, despite individual variation, the formula effectively captures the genetic contribution to height (8). In patients with precocious puberty, Quiroga et al. and Kagizmanli et al.—studying girls receiving gonadotropin-releasing hormone analog (GnRHa) therapy—both compared genetic target heights calculated with the Tanner method against results from bone-age prediction methods (Bayley-Pinneau, BP; Tanner-Whitehouse 2, TW2) to evaluate whether treatment enabled patients' final height to return within their genetic potential range (9, 10). In patients with growth hormone deficiency (GHD), Erbaş et al. similarly assessed the performance of the Tanner method alongside two other major prediction methods (11). These studies consistently used “the difference between final height and genetic target height” or “whether the target height range was attained” as the core outcome measure, underscoring the critical role of the Tanner method in clinical evaluation.

The Target Height Completion Rate (THCR) is defined as:

THCR = (measured adult height / Tanner-method genetic target height) × 100%.

As an index additionally adjusted for genetic background, THCR directly quantifies the degree to which an individual realizes their genetic height potential, supplementing the population-relative information provided by SDS. By integrating parental heights, THCR offers a complementary perspective for evaluating whether growth retardation reflects familial short stature or pathological processes, and provides a genetic-background-adjusted benchmark for assessing the efficacy of growth-promoting treatments. Establishing ethnicity-specific, population-based normal ranges of THCR can further support accurate quantification of the impacts of disease, nutrition and socioeconomic factors on height development.

### 1.3 Study objectives

Although the Tanner method has been used in clinical genetic counseling for several decades and THCR is a readily calculable indicator, its range and stability in the Chinese population have not been systematically investigated. We introduce THCR to examine its distributional characteristics, reference ranges, and cross-subgroup stability in the general population. Accurately assessing genetic target height completion based on the Tanner method in ordinary Chinese individuals and establishing normal THCR reference ranges are prerequisites for applying this indicator in disease cohort research.

Leveraging the large-scale, population-based China Health and Nutrition Survey (CHNS) cohort with 5,098 adults and complete parental height records, this study aims to: (1) establish population-level percentile reference ranges of THCR in general Chinese adults; (2) systematically evaluate the stability of THCR across key demographic subgroups, including sex, birth cohort, urban-rural residence and educational attainment; (3) verify the clinical application potential of THCR as a genetic-adjusted growth assessment tool, to provide evidence support for individualized growth evaluation and precision medicine practice.

## 2 Methods

### 2.1 Data source

This study used data from the China Health and Nutrition Survey (CHNS), an ongoing prospective cohort study conducted collaboratively by the National Institute for Nutrition and Health of the Chinese Center for Disease Control and Prevention (formerly the Institute of Nutrition and Food Hygiene, Chinese Academy of Preventive Medicine) and the Carolina Population Center at the University of North Carolina (12). Although the CHNS is a prospective cohort study, the present analysis used a cross-sectional design nested within the cohort. The longitudinal structure of the cohort was used for two specific purposes: (1) to ascertain adult status at the time of measurement, and (2) to reduce measurement error in parental heights by averaging across survey waves. The CHNS employed multistage random cluster sampling, covering urban and rural communities across nine provinces in eastern, central, and western China (Liaoning, Heilongjiang, Jiangsu, Shandong, Henan, Hubei, Hunan, Guangxi, and Guizhou). Data collection began in 1989, with follow-up surveys in 1991, 1993, 1997, 2000, 2004, 2006, 2009, 2011, and 2015, comprising ten survey waves. Previous work on height development using CHNS data has been published (13).

This study utilized publicly available CHNS data including family member relationships and parental ID records, demographic information (sex, year of birth), physical examination data, and educational attainment data.

### 2.2 Study population

Inclusion criteria: (1) identifiable biological parents; (2) complete physical examination height data; (3) age ≥18 years at the time of survey; (4) both parents having documented measured height (the Tanner target height formula requires heights of both parents).

Exclusion criteria: Extreme height outliers—offspring height < 120 cm or >210 cm, paternal height < 130 cm or >210 cm, maternal height < 120 cm or >200 cm. With reference to height distribution characteristics of the Chinese adult population (14), these cutoff thresholds are substantially wider than the normal physiological fluctuation range and serve solely to exclude obvious measurement errors and data entry mistakes, without introducing bias to the overall sample distribution.

Handling of parental height: For parents with multiple physical examination records, the cross-wave mean was used as the height estimate to reduce random error from single measurements.

Handling of offspring height: Given that some individuals aged 18 years may not have attained their final adult height, we adopted each participant's latest height measurement to obtain the closest approximation of their true final adult height.

### 2.3 Variable definitions

Genetic target height (Tanner method):

Male target height (cm) = (father's height + mother's height + 13) / 2.

Female target height (cm) = (father's height + mother's height – 13) / 2.

Target Height Completion Rate (THCR):

THCR = (measured adult height / Tanner-method target height) × 100%.

Subgroup analyses were pre-specified in this study, incorporating four variables: Sex (male, female); Birth cohort (categorized by year of birth: < 1950, 1950s, 1960s, 1970s, 1980s, 1990s); Urban-rural residence (urban, rural); Educational attainment (categorized by highest level attained: low [below junior high school], medium [junior high school / senior high school / technical secondary school], high [college/university and above]). These variables were selected *a priori*, as they collectively represent the core biological and environmental factors affecting height. Sex is the primary biological determinant and serves as the foundation of the sex-specific Tanner target height formula. Birth cohort was included to explore whether the prominent secular trend of increasing height among Chinese populations exerts an impact on Target Height Completion Rate (THCR). Urban-rural residence and educational attainment are indispensable proxies for the socioeconomic and nutritional environments that shape final adult height. The objective of these subgroup analyses was to verify whether a unified population-wide THCR reference range is applicable to diverse social subgroups.

### 2.4 Statistical analysis

Per official documentation, the CHNS does not provide sampling weights; all analyses were conducted without weighting. All data processing and analysis were performed using Python (pandas, numpy, matplotlib). Continuous variables are expressed as mean ± standard deviation (Mean ± SD), and categorical variables as frequencies (percentages). Between-group comparisons employed independent samples *t*-tests (two groups) or one-way analysis of variance (three or more groups). Percentile ranges (P1–P99) were used to describe the distributional characteristics of THCR in the general population. The significance level was set at α = 0.05 (two-tailed).

To evaluate the robustness of the reference ranges to the sex-difference constant assumed by the Tanner formula, we computed the sex difference in height within the CHNS (for both the parental and offspring generations) and recomputed THCR using a population-specific constant based on the observed difference.

To examine the association between THCR and each factor while simultaneously adjusting for the others, we additionally fitted a multivariable linear regression including sex, birth cohort, urban–rural residence, and educational attainment; results are reported as adjusted coefficients (percentage points) with 95% confidence intervals.

To display between-subgroup variation, figures use truncated y-axes; the exact axis range is stated in each figure legend.

### 2.5 Ethical approval

The CHNS project received ethical approval from the institutional review boards of the National Institute for Nutrition and Health, Chinese Center for Disease Control and Prevention, and the University of North Carolina. The present study constitutes a secondary analysis of publicly available data and does not require additional ethical approval.

## 3 Results

### 3.1 Study population characteristics

A total of 5,098 adult individuals with complete matched parental height records were included (each contributing only the most recent height measurement). Of these, 3,122 (61.2%) were male and 1,976 (38.8%) female. Urban residents accounted for 36.8% (*n* = 1,875) and rural residents for 63.2% (*n* = 3,223). Educational attainment was predominantly at the medium level (70.6%), with the low education group constituting 15.8% and the high education group 12.6% (Table 1).

**Table 1:** Population characteristics.

Variable	Total (*N* = 5,098)	Males (*n* = 3,122)	Females (*n* = 1,976)
Offspring height, cm	164.6 ± 8.4	168.9 ± 6.2	157.9 ± 6.0
Paternal height, cm	165.1 ± 6.3	165.1 ± 6.3	165.0 ± 6.3
Maternal height, cm	154.3 ± 6.1	154.3 ± 6.0	154.4 ± 6.1
Tanner target height, cm	161.1 ± 7.9	166.1 ± 5.0	153.3 ± 4.9
THCR, %	102.19 ± 3.25	101.69 ± 3.13	102.99 ± 3.28
Urban residence, %	36.8	35.2	39.3
Higher education, %	12.6	11.3	14.6

### 3.2 Overall distribution of THCR

The overall population mean THCR was 102.19% (SD 3.25%), with a median of 102.18%, conforming to an approximately normal distribution. The full percentile distribution is presented in Table 2. All between-subgroup differences were ≤ 1.5 percentage points among cohorts from the 1950s onward; the population-level P5 (5th percentile) was 97.03% and P2.5 (2.5th percentile) was 95.84%. These results suggest that THCR exhibits favorable cross -subgroup stability in the general Chinese population.

**Table 2:** THCR95% confidence intervals.

Group	Overall (95% CI)	Male (95% CI)	Female (95% CI)
N	5,098	3,122	1,976
Mean	102.19 (102.10, 102.28)	101.69 (101.58, 101.79)	102.99 (102.85, 103.13)
SD	3.25 (3.16, 3.33)	3.13 (3.03, 3.22)	3.28 (3.13, 3.43)
P1	94.25 (93.71, 94.73)	93.87 (93.25, 94.57)	95.05 (94.40, 95.71)
P2.5	95.84 (95.57, 96.12)	95.46 (95.10, 95.89)	96.69 (96.02, 97.16)
P5	97.03 (96.86, 97.33)	96.68 (96.40, 96.94)	97.77 (97.49, 98.21)
P10	98.24 (98.10, 98.36)	97.90 (97.71, 98.06)	99.18 (98.92, 99.45)
P25	100.15 (100.04, 100.26)	99.67 (99.51, 99.84)	100.97 (100.80, 101.13)
P50	102.18 (102.06, 102.28)	101.71 (101.61, 101.83)	102.91 (102.75, 103.05)
P75	104.24 (104.13, 104.37)	103.77 (103.59, 103.92)	105.08 (104.86, 105.23)
P90	106.19 (105.98, 106.39)	105.58 (105.46, 105.76)	107.09 (106.81, 107.33)
P95	107.36 (107.17, 107.59)	106.67 (106.42, 106.86)	108.43 (108.15, 108.76)
P97.5	108.61 (108.34, 108.90)	107.44 (107.14, 107.97)	109.43 (109.11, 109.77)
P99	109.88 (109.59, 110.10)	109.12 (108.38, 109.65)	110.51 (109.99, 111.23)

95% confidence intervals for all estimates were obtained by nonparametric bootstrap resampling (2,000 replicates).

### 3.3 Birth cohort trends

Variation in THCR across birth cohorts was minimal: 102.03% for the 1950s, 102.05% for the 1960s, 102.17% for the 1970s, 102.33% for the 1980s, and 102.35% for the 1990s. For the cohorts from the 1950s onward, cohort means all fell within the narrow band of 102.0%−102.4%. During this same period, offspring absolute height exhibited a pronounced secular increase, suggesting that parental heights embedded in the Tanner target height increased concurrently, thereby preserving cross-generational stability of the ratio. These findings are consistent with previous research (15–17) (Figure 1).

**Figure 1:**
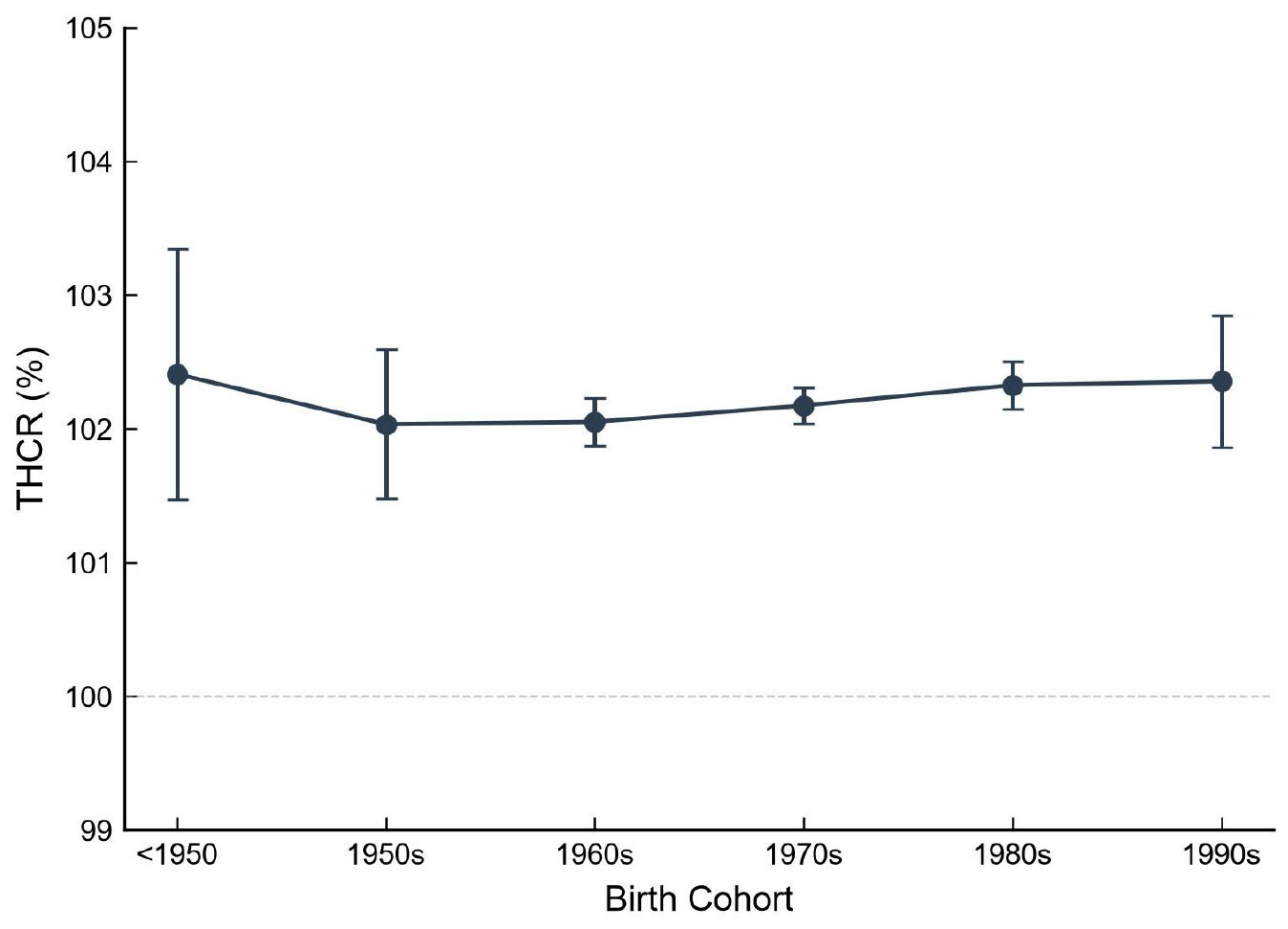
Y-axis truncated to [99, 105] to display between-group variation.

The apparent flatness was not an artifact of the combined sample: sex-specific trends within the 1950s−1990s cohorts were also flat, with male THCR ranging from 101.5% to 101.9% and female THCR from 102.8% to 103.4% (Figure 2). Female THCR was visibly higher in the two earliest cohorts (104.3% for < 1,950, *n* = 9; 103.4% for the 1950s, *n* = 25) before declining to a stable plateau. Given the very small numbers of female participants in these cohorts and Its sampling error bars nearly overlap the full range of error bars for all subsequent years, the data do not permit a reliable distinction between a genuine early-cohort phenomenon and sampling variability, and no specific mechanism is therefore proposed. The pattern was female-specific: no corresponding elevation was observed among males (< 1,950: 101.8%, *n* = 30). From the 1960s cohort onward—comprising 98% of female participants—the mean THCR was flat (females 102.8%−103.1%; males 101.5%−101.9%).

**Figure 2:**
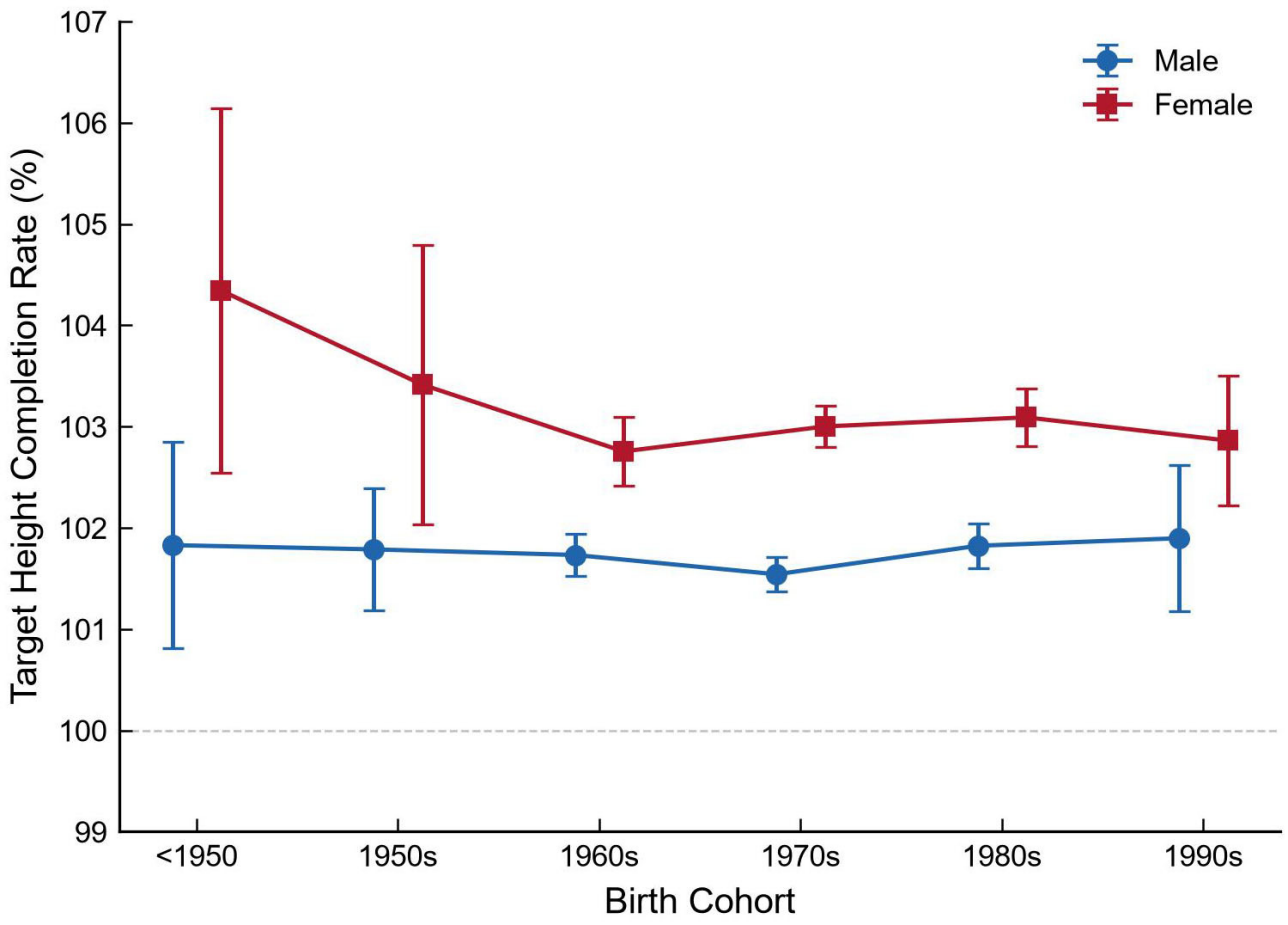
Points are cohort means; error bars are 95% confidence intervals. The y-axis is truncated to [99, 107] % to display between-subgroup variation; all cohort means lie within approximately 101.5%−104.3%, so a full-scale axis would visually compress these differences. Female THCR was higher in the two earliest cohorts (104.3%, *n* = 9; 103.4%, *n* = 25), whose confidence intervals overlap those of subsequent cohorts; from the 1960s cohort onward, female means were flat (102.8%−103.1%) and male means were flat (101.5%−101.9%).

### 3.4 Sex differences

THCR exhibited a significant sex difference: females (102.99% ± 3.28%) exceeded males (101.69% ± 3.13%), *P* < 0.001, with a mean difference of approximately 1.30 percentage points. This disparity may partly reflect a systematic bias between the sex correction constant (±13 cm) in the Tanner formula and the actual sex-specific height difference in the contemporary Chinese population: if the real male-female height gap is less than 13 cm, the Tanner formula would systematically overestimate male target height or underestimate female target height, thereby generating a sex difference in THCR. A systematic analysis based on five rounds of the China National Nutrition Survey (1982–2015) reported that by 2015, the mean height of Chinese adults aged 18–44 years was 169.7 cm for males and 158.0 cm for females, a difference of 11.7 cm (18), corroborating this systematic bias. Figure 2. Cohort trends in THCR stratified by sex. Females exceeded males across all birth cohorts.

The measured sex difference in height in the CHNS was 10.8 cm in the parental generation and 11.0 cm in the offspring generation, both smaller than the 13 cm assumed by the Tanner formula. When THCR was recomputed using the observed population-specific constant (±11 cm), the sex difference in THCR essentially disappeared (1.30 → 0.02 percentage points, *P* = 0.86), whereas the overall reference values were essentially unchanged (Table 3).

**Table 3:** THCR recomputed with a population-specific sex constant (±11 cm, based on the observed male–female height difference in the CHNS) to test robustness to the Tanner formula's ±13 cm constant.

Statistic	Tanner (+-13 cm)	Population-specific (+-11 cm)	Difference (pp)
*N*	5,098.00	5,098.00	0
Mean	102.19	102.31	0.12
SD	3.25	3.19	−0.06
Median	102.18	102.30	0.12
P1	94.25	94.39	0.14
P2.5	95.84	96.03	0.19
P5	97.03	97.18	0.15
P10	98.24	98.50	0.26
P25	100.15	100.29	0.14
P50	102.18	102.30	0.12
P75	104.24	104.40	0.16
P90	106.19	106.26	0.07
P95	107.36	107.48	0.12
P97.5	108.61	108.40	−0.21
P99	109.88	109.78	−0.10
Male mean	101.69	102.30	0.62
Female mean	102.99	102.32	−0.67
Sex difference (pp)	1.30	0.02	−1.28
Sex difference *P*	<0.001	0.86	−

### 3.5 Urban–rural differences

Urban residents exhibited a slightly higher THCR (102.38% ± 3.22%) than their rural counterparts (102.08% ± 3.26%), *P* = 0.002, with a difference of approximately 0.30 percentage points. This urban–rural disparity was substantially smaller than the sex difference and may be mediated by divergent socioeconomic conditions and nutritional status between urban and rural settings (Figure 3).

**Figure 3:**
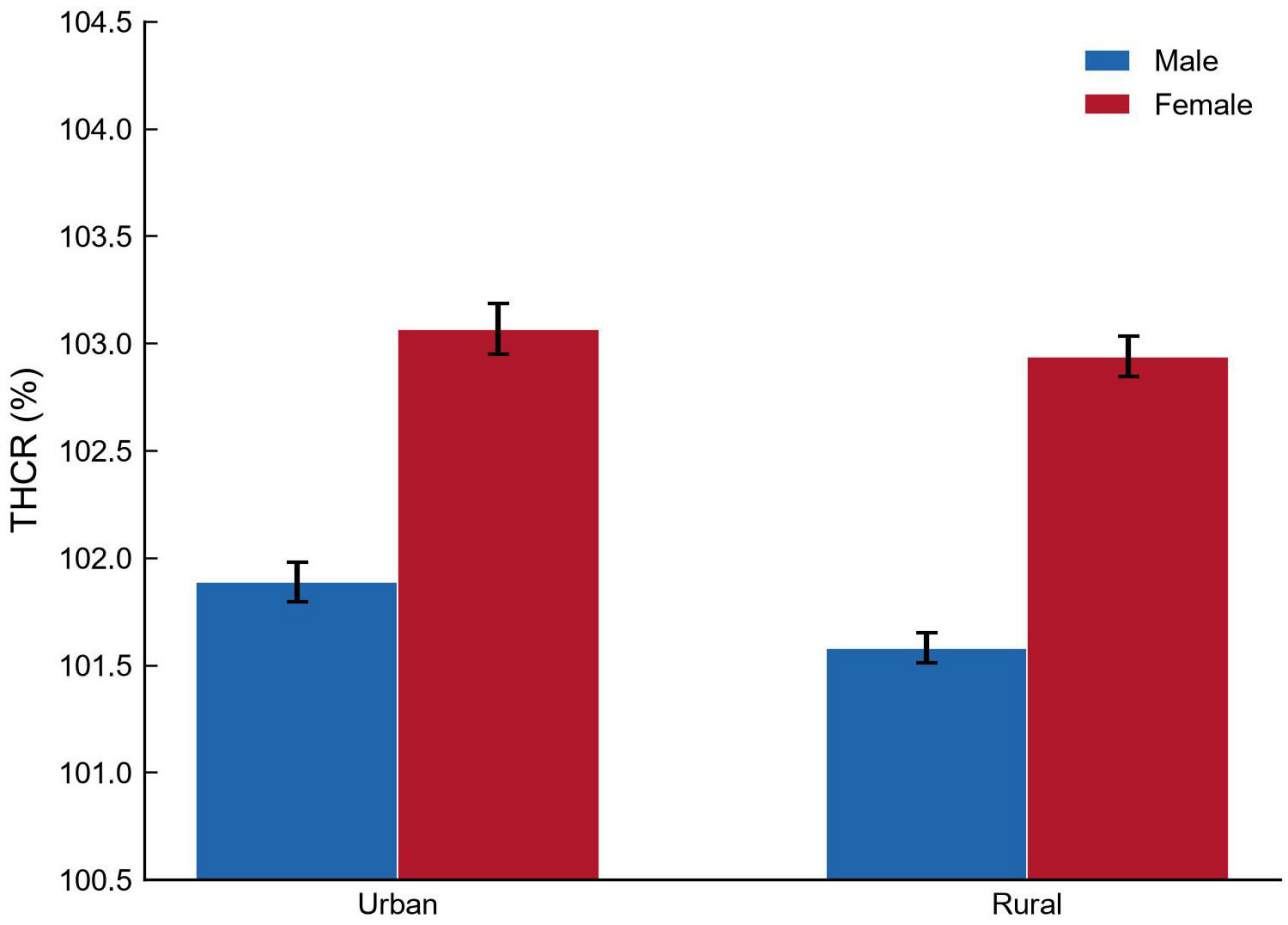
Y-axis truncated to [100.5, 104.5] to display between-group variation.

### 3.6 Educational attainment

THCR exhibited a graded increase with educational attainment: 101.87% ± 3.67% in the low education group (below junior high school), 102.19% ± 3.19% in the medium education group, and 102.67% ± 2.86% in the high education group (college or above). The difference between the low and high education groups was statistically significant (*P* < 0.001) Figure 4.

**Figure 4:**
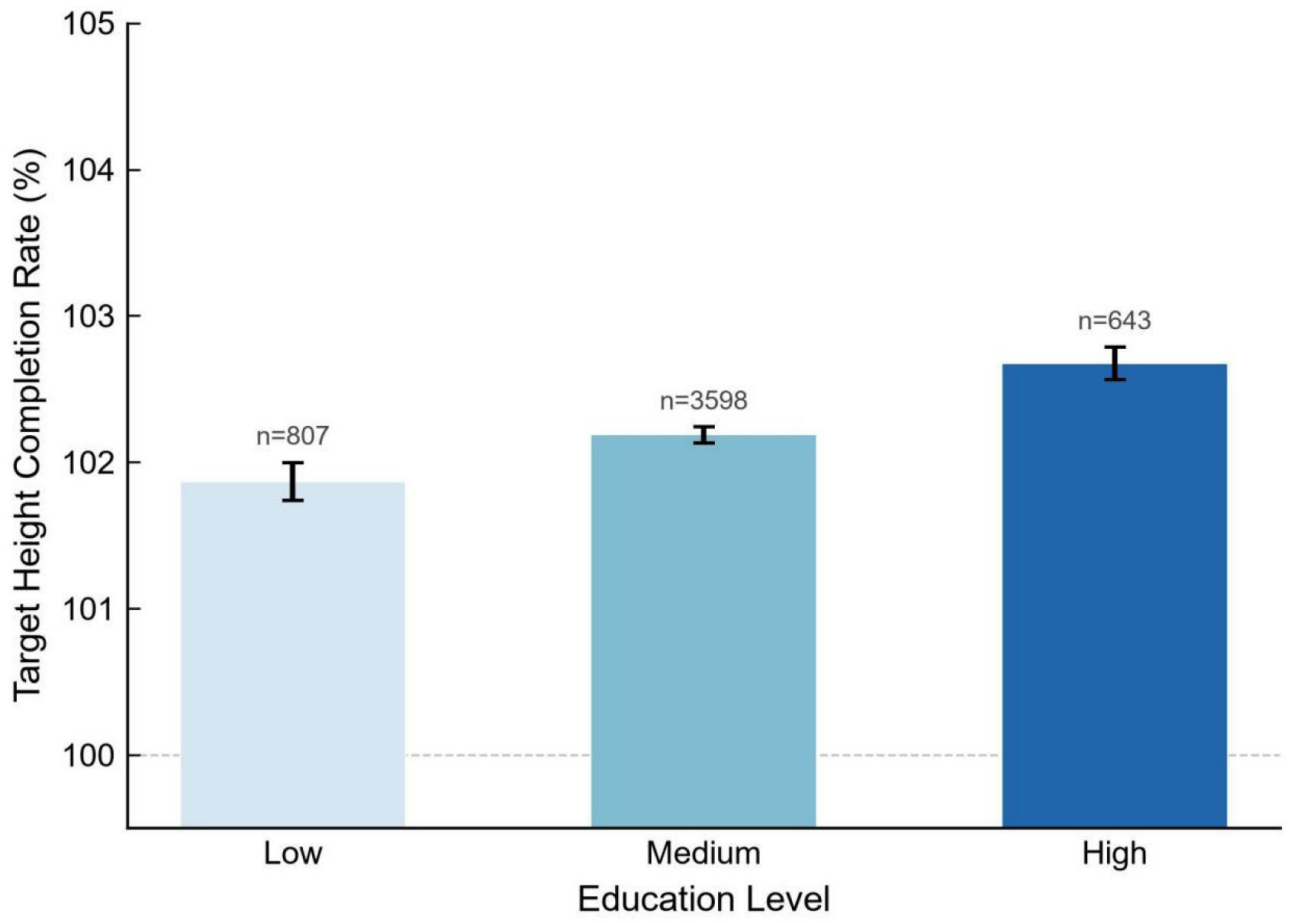
Y-axis truncated to [99.5, 105] to display between-group variation.

After sex stratification, the educational gradient was primarily observed in males (low: 101.27%, medium: 101.67%, high: 102.45%; low vs. high, *P* < 0.001), whereas among females, educational attainment showed no significant association with THCR (low: 102.69%, medium: 103.06%, high: 102.95%; all pairwise comparisons, *P* > 0.05) Figure 5.

**Figure 5:**
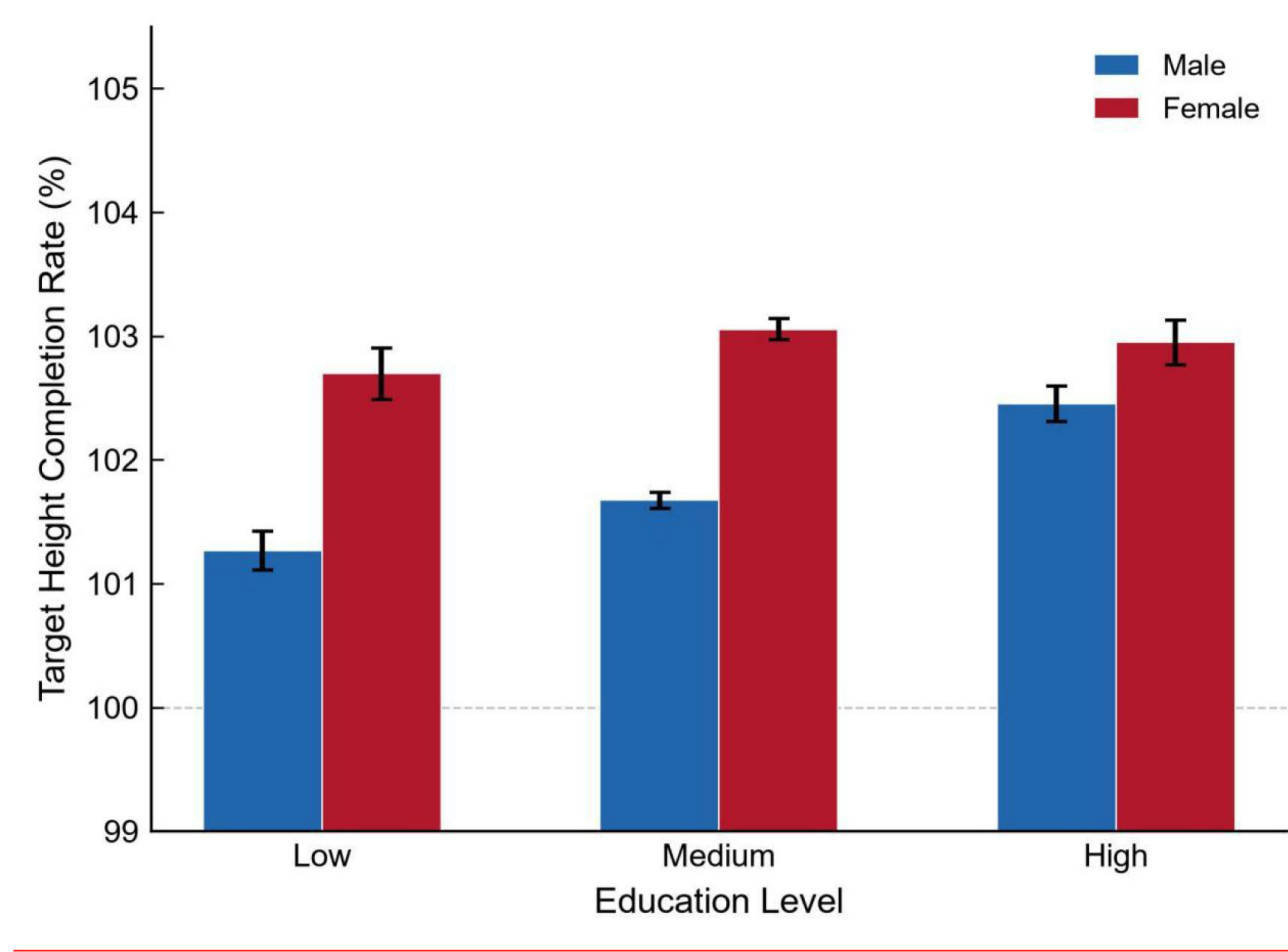
THCR of males and females across different education levels.

### 3.7 Multivariable analysis

Table 4 presents the association of THCR with each factor after simultaneous adjustment for the others. Reference categories were male sex, the 1950s birth cohort, rural residence, and low educational attainment. Female sex was associated with a higher THCR (+1.28 pp, 95% CI 1.10–1.46, *P* < 0.001), and THCR increased with educational attainment (medium vs. low: +0.36 pp, 95% CI 0.12–0.61, *P* = 0.004; high vs. low: +0.69 pp, 95% CI 0.34–1.04, *P* < 0.001). In contrast, the urban–rural difference was attenuated and no longer statistically significant after adjustment (+0.14 pp, 95% CI −0.05 to 0.33, *P* = 0.156), indicating that the univariable urban–rural difference largely reflected the higher educational attainment of urban residents. Birth cohort was not significantly associated with THCR (overall *P* = 0.68).

**Table 4:** Multivariable linear regression of THCR on sex, birth cohort, urban–rural residence, and educational attainment. Reference categories: male sex; 1950s birth cohort; rural residence; low educational attainment.

Factor	Coefficient (pp)	95% CI	*P*-value
Female (vs. male)	1.28	[1.10, 1.46]	<0.001
1960s (vs. 1950s)	−0.23	[−0.76, 0.29]	0.384
1970s	−0.30	[−0.81, 0.22]	0.26
1980s	−0.19	[−0.71, 0.34]	0.482
1990s	−0.24	[−0.95, 0.46]	0.5
<1950	0.32	[−0.80, 1.43]	0.576
Urban (vs. rural)	0.14	[−0.05, 0.33]	0.156
High education (vs. low)	0.69	[0.34, 1.04]	<0.001
Medium education (vs. low)	0.36	[0.12, 0.61]	0.004

## 4 Discussion

### 4.1 Main findings

Using a large sample from the China Health and Nutrition Survey (CHNS), this study systematically characterized the distribution of target height completion rate (THCR) in a general Chinese population. The principal findings are as follows.

First, the mean THCR in general Chinese adults was approximately 102.19% (SD 3.25%), indicating that attained adult height modestly exceeds the Tanner-predicted genetic target height by roughly 2%. This small “excess” may reflect a positive secular shift attributable to improvements in nutrition and living standards over recent decades.

Second, THCR demonstrated strong cross-subgroup stability. Regardless of stratification by birth cohort, urban–rural residence, or educational attainment, among the cohorts from the 1950s onward, subgroup means ranged between 101.7% and 103.0%, with the maximum between-group difference not exceeding 1.5 percentage points. This stability underscores the indicator's potential utility for clinical decision-making.

Third, the multivariable analysis indicated that the sex and educational gradients persisted after simultaneous adjustment for the other factors (female vs. male: +1.28 pp, 95% CI 1.10–1.46, *P* < 0.001; high vs. low education: +0.69 pp, 95% CI 0.34–1.04, *P* < 0.001), whereas the univariable urban–rural difference was attenuated and no longer statistically significant after adjustment (+0.14 pp, 95% CI −0.05 to 0.33, *P* = 0.156), suggesting that the apparent urban–rural difference may reflected the higher educational attainment of urban residents.

### 4.2 Clinical significance of genetic background adjustment

Height SDS, by its nature, compares an individual against the population mean. This approach has inherent limitations when confronted with conditions such as familial short stature (FSS). Children with FSS typically present with a height below −2 SDS (or below the 3rd percentile) relative to age- and sex-matched healthy peers; nonetheless, they maintain a stable, normal growth velocity consistent with their genetic potential (19), because their short stature stems from a low genetic target height rather than from an underlying pathological condition. Standard SDS evaluation alone cannot adequately distinguish this clinical scenario, as SDS does not account for individual differences in genetic target height.

It should be emphasized that the standard deviation score (SDS) remains a well-validated and effective screening tool for identifying growth abnormalities. Importantly, THCR and SDS are not interchangeable but complementary, as they reflect different dimensions of information. SDS reflects “relative position within the population,” whereas THCR reflects “degree of realization of genetic potential,” offering a more individualized assessment aligned with the goals of personalized medicine. The combined use of both indicators may yield more comprehensive information than either metric alone. The establishment of THCR reference range provides a quantitative tool for individualized growth assessment, which is an important supplement to the population-based SDS system and conforms to the development trend of precision medicine in pediatric growth and development.

### 4.3 Revisiting sex differences

We observed that female THCR exceeded male THCR by approximately 1.30 percentage points. This discrepancy likely originates primarily from the intrinsic characteristics of the Tanner formula rather than from genuine biological differences. The sex correction constant of ±13 cm in the Tanner formula was derived from 1970 British population data (7). In the contemporary Chinese adult population, the mean male–female height difference is approximately 10–12 cm, which is smaller than 13 cm. When the actual sex-specific height gap is less than the correction constant embedded in the Tanner formula, the formula will systematically overestimate male target height (thus suppressing male THCR) or underestimate female target height (thus inflating female THCR). This interpretation is supported by a sensitivity analysis (Section 3.4): recomputing THCR with the observed CHNS sex difference (± 11 cm) reduced the sex difference in THCR from 1.30 to 0.02 percentage points (*P* = 0.86), confirming that the sex difference is attributable to the fixed ± 13 cm constant of the Tanner formula rather than genuine biological differences. These considerations counsel caution in cross-sex comparisons and support the practice of reporting THCR stratified by sex.

### 4.4 Limitations

Several limitations of this study warrant acknowledgment. First, the CHNS employs a multistage cluster sampling design covering nine provinces. While it offers substantial geographic and economic diversity, it lacks full national representativeness (certain regions of northwestern and southwestern China are not covered), and no relevant data are available for other countries, regions, or ethnic groups. Consequently, the results presented herein should be regarded as reference values for the CHNS-covered population, with national representativeness requiring validation through larger-scale surveys.

Second, this study did not specifically exclude individuals with certain conditions known to influence linear growth, such as chronic kidney disease or growth hormone deficiency. Nevertheless, the population prevalence of these disorders is very low, and the CHNS dataset does not contain records of such conditions, which limits our ability to perform such exclusions.

Third, the prediction interval of the Tanner formula is relatively wide (approximately ± 8.5 cm), implying considerable individual-level variation in THCR. Interpretations for individuals near the boundaries of the reference range should therefore be made with caution. We recommend that clinicians interpret individual THCR values in the context of longitudinal growth velocity, bone age, and other clinical findings. The percentile-based reference ranges established in this study serve as population-level statistical benchmarks rather than precise individual prediction tools.

Fourth, parental heights were measured in adulthood; height loss due to intervertebral disc degeneration in some older parents may introduce minor measurement error, though this is expected to be negligible at the population level (20, 21).

Fifth, the Tanner formula was derived from a 1970 European population. The finding of a mean THCR around 102% in this study suggests acceptable population-level applicability in the Chinese context, yet the cross-ethnic generalizability of the formula itself warrants further evaluation. Because parental height also reflects the parents' growth environment, THCR does not isolate purely genetic height potential; the mean THCR of ~102% likely reflects, in part, the modest intergenerational height gain attributable to secular improvements in nutrition and living conditions.

Sixth, the analytic sample was male-predominant (61.2% males vs. 38.8% females). In principle, this could affect the pooled estimates in two ways: the pooled THCR and its reference range are sex-composition-weighted (male THCR 101.69% vs. female 102.99%), and unequal group sizes could reduce precision for the smaller female subgroup.

Seventh, while unweighted analysis is a common practice in existing publications based on the CHNS dataset, the absence of sampling weights in our analysis may introduce bias to all statistical estimates. We will pursue this research question using a more appropriate database in future studies.

Finally, because the CHNS samples multiple family members per household, the study population includes siblings and, in some cases, multiple generations from the same household. Analyses treated individuals as independent observations, and family-level clustering was not explicitly modeled; this may affect the precision of the standard errors.

### 4.5 Future directions

The THCR in the present study was defined based on adult final height; therefore, the derived adult reference range cannot be directly applied to children who are still in the growth and development period. Future research may proceed along the following lines: (1) measuring THCR in disease cohorts known to affect height—such as chronic kidney disease (CKD), congenital heart disease (CHD), and growth hormone deficiency (GHD)—to validate its sensitivity as an outcome measure and compare its performance against SDS; previous studies have reported that growth retardation affects 30%−50% of children with CKD, with prevalence closely related to disease severity (22, 23), and that children with CHD, owing to high energy expenditure, chronic hypoxia, feeding difficulties, and recurrent hospitalization, exhibit a significantly higher incidence of growth failure than healthy controls (24, 25); (2) developing age-specific THCR reference curves for children and adolescents, enabling the indicator to serve an early warning function in growth monitoring; (3) leveraging the approximately 2,000 CHNS participants with childhood dietary records to explore associations between childhood nutritional exposure and adult THCR; (4)In the current era of rapid progress in artificial intelligence, the integration of AI-driven analytical techniques holds promise for refining the evaluation of genetic potential completion in individuals as well as in populations, thereby achieving greater precision than conventional statistical methods (26).

## 5 Conclusions

Among general Chinese adults, the mean THCR derived using the Tanner method was 102.19% (SD 3.25%), with P5 at 97.03% and P2.5 at 95.84%. This indicator demonstrated favorable cross-group stability across sex, birth cohort, urban–rural residence, and educational attainment strata. The percentile distribution established in this study may serve as a baseline reference for future disease cohort investigations.

## Data Availability

Publicly available datasets were analyzed in this study. This data can be found here: https://dataverse.unc.edu/dataverse/chns.
